# Assisted PD throughout Europe: advantages, inequities, and solution proposals

**DOI:** 10.1007/s40620-023-01765-y

**Published:** 2023-10-19

**Authors:** Anabela Malho Guedes, Sally Punzalan, Edwina A. Brown, Agneta Ekstrand, Maurizio Gallieni, Maite Rivera Gorrín, Helga Gudmundsdottir, Marco Heidempergher, Benno Kitsche, Thierry Lobbedez, Ulrika Hahn Lundström, Kate McCarthy, George J. Mellotte, Olivier Moranne, Dimitrios Petras, Johan V. Povlsen, Martin Wiesholzer

**Affiliations:** 1 Serviço de Nefrologia, Centro Hospitalar Universitário Do Algarve, Faro, Portugal; 2 Imperial College Renal and Transplant Centre, Imperial College Healthcare NHS Trust, London, UK; 3 Abdomen Centre, Nephrology Helsinki University Hospital, Helsinki, Finland; 4 Department of Biomedical and Clinical Sciences, Università Di Milano, Milan, Italy; 5 Nephrology and Dialysis Unit, ASST Fatebenefratelli Sacco, Milan, Italy; 6 Servicio de Nefrología, Hospital Ramón Y Cajal, Madrid, Spain; 7 Department of Nephrology, Oslo University Hospital, Ulleval, Oslo Norway; 8 Kuratorium Für Dialyse Und Nierentransplantation E.V, Cologne, Germany; 9 NADia—Netzwerk assistierte Dialyse, Berlin, Germany; 10 Néphrologie, CHU CAEN, Normandy, France; 11 Division of Renal Medicine, Department of Clinical Science, Intervention and Technology, Karolinska Institutet, Stockholm, Sweden; 12 Baxter Healthcare Ltd, Wallingford, Compton, Newbury UK; 13 Trinity Health Kidney Centre, Tallaght University Hospital, Tallaght, Dublin, Ireland; 14 Department of Nephrology-Dialysis-Apheresis, CHU Caremeau Nimes, IDESP Montpellier University, Nimes, France; 15 Department of Nephrology, General Hospital ‘Hippokration’, Athens, Greece; 16 Department of Renal Medicine, Aarhus University Hospital, Aarhus, Denmark; 17 Clinical Department for Internal Medicine, University Hospital St Poelten, Karl Landsteiner University of Health Sciences, St Poelten, Austria

**Keywords:** Assisted Peritoneal Dialysis, Community care, Frailty, Equity, Quality of life

## Abstract

**Background:**

Peritoneal dialysis provides several benefits for patients and should be offered as first line kidney replacement therapy, particularly for fragile patients. Limitation to self-care drove assisted peritoneal dialysis to evolve from family-based care to institutional programs, with specialized care givers. Some European countries have mastered this, while others are still bound by the availability of a volunteer to become responsible for treatment.

**Methods:**

A group of leading nephrologists from 13 European countries integrated real-life application of such therapy, highlighting barriers, lessons learned and practical solutions. The objective of this work is to share and summarize several different approaches, with their intrinsic difficulties and solutions, which might helpperitoneal dialysis units to develop and offer assisted peritoneal dialysis.

**Results:**

Assisted peritoneal dialysis does not mean 4 continuous ambulatory peritoneal dialysis exchanges, 7 days/week, nor does it exclude cycler. Many different prescriptions might work for our patients. Tailoring PD prescription to residual kidney function, thereby maintaining small solute clearance, reduces dialysis burden and is associated with higher technique survival. Assisted peritoneal dialysis does not mean assistance will be needed permanently, it can be a transitional stage towards individual or caregiver autonomy. Private care agencies can be used to provide assistance; other options may involve implementing PD training programs for the staff of nursing homes or convalescence units. Social partners may be interested in participating in smaller initiatives or for limited time periods.

**Conclusion:**

Assisted peritoneal dialysis is a valid technique, which should be expanded. In countries without structural models of assisted peritoneal dialysis, active involvement by the nephrologist is needed in order for it to become a reality.

**Graphical abstract:**

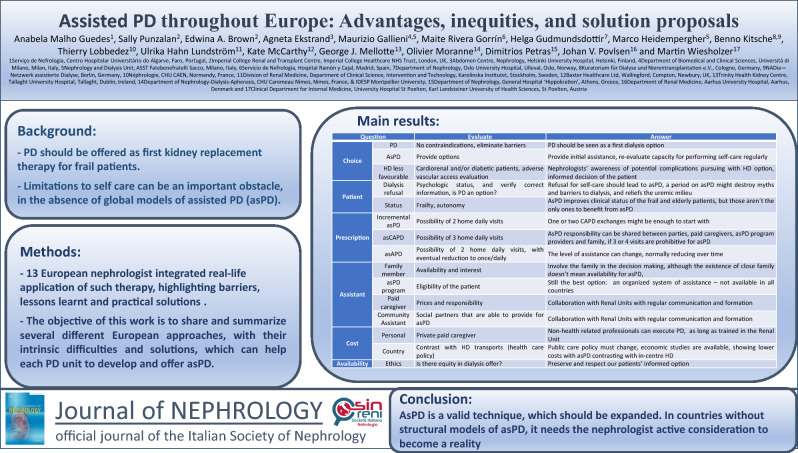

## Introduction

Peritoneal dialysis (PD) provides several benefits for patients and should be offered as the first kidney replacement therapy when pre-emptive kidney transplant is not feasible, particularly for fragile patients [1]. Over the years, numerous barriers hindering PD initiation have been identified. As barriers have arisen, solutions appear in the same proportion of the imagination and resources of the nephrologist. Limitations to self-care drove assisted peritoneal dialysis (asPD) to evolve from family-based care to institutional programs, with specialized care givers. Since the first report of asPD was published in Europe [2], things have evolved, but important inequities in healthcare systems still persist. Although asPD has been available and widely used in some European countries for many years now, it remains unavailable or poorly utilized in many others [3]. Some European countries have overcome this, while others are still bound by the availability of volunteers to become responsible for treatment, otherwise, in-center hemodialysis (HD) becomes inevitable. A recent survey among European nephrologists revealed that around 40% of PD centers had no access to asPD [4]. In a recent paper requesting a call to action for increased and equal access to PD based on Assisted PD, a group of leading nephrologists from several western European countries formed a group to drive increased availability of asPD in Europe and in their own countries [3]. From this collaboration, insights were provided into the main advantages and obstacles to PD for older, frail people; different global models of assisted PD delivery were compared, and the experience of asPD across 13 European countries was shared.

In this context, the same panel of specialists amalgamated real-life application of such therapy, highlighting barriers, lessons learned and practical solutions. Each experience is unique, but each one of them is focused in providing the best care to a non-autonomous patient for PD technique. The objective of this work is to share and summarize several different European approaches, with their intrinsic difficulties and solutions, which might help each PD unit to develop and offer asPD on a regular basis.

## Methods

Against this background, some of the leading nephrologists from several western European countries formed a group to drive increased availability of asPD in Europe and in their own countries. This panel included one or two nephrologists/country, from 13 different countries. The meetings started in 2021 in response to the need to increase access to asPD across Europe. These meetings were virtual, timetabled and sponsored by Baxter Healthcare (Europe). As part of this initiative, each member of the group provided information about the use of asPD in their own center and country, barriers to further developments and priorities to enable expansion of access to asPD. Each panel member was asked to contribute with a case report to illustrate barriers, advantages, lessons learned and practical solutions, etc. These illustrative examples were compiled based on the different strategies that might augment asPD availability.

## Results

We describe 13 representative cases (Table1) and profiles (Table 2) for assisted PD in some countries in Western Europe. Even though healthcare systems are different and there are different social and cultural approaches, we can define some shared aspects that allow us to determine similar causes, circumstances and possible solutions for patients with end-stage kidney disease (ESKD).

**Table 1:** European Case Studies

Country	Problem	Action	Impact	Points of learning
Case study 1 Austria	71 years, on PD since 2011 Pleuroperitoneal leak in 2014 + psychiatric issues Relapse of leak after 8 weeks on HD Successful surgical repair Patient wants to stay on PD–no family support, lost ability for self-care Transition for HD was a major concern due to his psychiatric condition	Home based 24-h-care was needed for multiple issues APD regime, with 2 home visits by nurses of the Renal Unit Patient improved and learnt how to disconnect–asPD with 1 daily visit (for cycler set-up)	The patient maintained a home-base therapy, which enabled him to visit his sister, who was trained for APD, to ensure his short visits The patient gained social and health status, and acquired partial responsibility for his treatment	Interaction with his family (sister), his family doctor and the home based 24 h-care-assistants enabled the recovery of this patient In 2014 only specialized nurses in Nephrology were allowed to perform PD, resources were limited, 2-visit/day was the initial compromise
Case study 2 Finland	88 years, CKD Prevalent patient in APD Dementia ensued 5 years after PD initiation	Switched to CAPD 3times per day, PD staff educated the home care staff (that she was able to afford)	Quality of life maintained, avoiding travelling to a HD unit	Collaboration of Renal Units with private carers is a feasible option, when the patient has economic ability to pay, in the absence of a public care policy for asPD
Case study 3 France	85 years, CKD The patient chose PD, but unable to perform the technique due to cognitive dysfunction and dexterity His wife refused to assist him on the dialysis	Nurse-assisted CAPD, 3 exchanges per day was initiated	Improved quality of life, as he opted for asAPD and after 3 months he is able to connect and disconnect his PD catheter, assistance is maintained for cycler set-up	The existence of a close family member doesn’t mean availability for asPD The best option is an organized system of assistance The level of assistance can change, normally reducing over time
Case study 4 Italy	85 years, CKD stage 5 No family support, autonomous, but assisted by a caregiver in daily house routines The patient refused HD, refused self-care, opted initially for conservative treatment	AsPD was offered, and the patient agreed Caregiver was trained on PD The patient was started on incremental CAPD	The patient didn’t feel limited in his daily life, for 3 years, which was the main motif for refusal of dialysis in first place He travelled for summer vacation with his caregiver Dialysis was discontinued due to dementia	HD refusal must drive the team to offer PD, refusal for self-care led to asPD PD can be executed by non-health related professionals, as family members, or another caregiver
Case study 5 Ireland	80 years, frail, unable to perform PD, developing recurrent infections	Engaged a private healthcare nursing service	Remains on asPD, at home	Public care policy must change to support asPD similar to the support offered to pay two-way travel to HD facilities and higher HD costs, 3 times per week
	80 years, frail, unable to perform PD, developing recurrent infections	Limited family support Transferred to in-centre HD	In-centre HD, 3 times per week, with paid transportation	
Case study 6 Greece	78 years, cardiorenal syndrome, reduced vision No family support, 50 km from Renal Unit Patient refused HD	A caregiver (paid by the patient) was trained on PD Incremental asCAPD was initiated	The patient quality of life improved, hospital readmissions due to fluid overload ceased	No AsPD available, if no family member involved This was the only case of asPD (excluding supported by family member) in the last 25 years
Case study 7 Portugal	77 years, prevalent PD patient, well adapted to APD Suffers an ischemic cerebellar stroke–requires functional capacitation at a Physical Rehabilitation Unit Refusal of transition to HD, due to very low BP and bedridden state	Daughter offers to perform asAPD at home and to pay for rehabilitation sessions, as the Physical Rehabilitation Unit doesn’t provide PD support	The patient had both treatments at home–asAPD and rehabilitation Maintained preserved communication and cognitive status, previous functional state was not recovered	Equity in dialysis offer must be a priority to preserve our patients’ freedom of choice in Health Units (HD would be guaranteed during the Physical Rehabilitation Unit, relying on the transfer of a bedridden frail patient 3x/week, but asPD not considered)
Case study 8 Spain	62 years, cardiorenal syndrome, hypotensive, CKD PD proposed by his Cardiologist, as an ultrafiltration method	The spouse was trained on PD A nocturnal exchange was prescribed As the patient improved, he was autonomous for PD and most daily activities	Increased survival, this patient was on PD for 6 years Increased his quality of life, allowing him to drive again, and travel	Initial need of assistance, doesn’t mean the patient won’t become capable of performing self-care
Case study 9 Spain	78 years, blind, CKD Started HD, due to her blindness Multiple vascular access failures (5 AVF, 4 tunnelled CVC, 5 provisory catheters, from 2001 to 2004), nephrologist preference for HD could have been a barrier to earlier transferal to PD	The spouse was trained on PD The patient was on asPD for 31 months Died due to pneumonia	PD decreased her and her husband anxiety towards vascular access interventions PD decreased associated costs	Vascular access planification could have predicted such difficulties and asPD could have been the first dialysis option PD should be seen as a first dialysis option, if absence of self-care possibility. assisted PD must be addressed by health care teams
Case study 10 Spain	19 years, cerebral palsy, CKD The parents opted for PD	The parents were trained on PD APD with dry day was prescribed	PD allowed her to stay home with her parents, avoiding 3x/week travel to HD centre	Not only frail and elderly patients benefit from asPD
Case study 11 Sweden	73 years, diabetic, high BP, CKD, amputation of the right lower leg, bedridden in the post-op of lower left leg amputation	Advanced health care team offered to provide asPD The patient refused APD, due to noise and alarms CAPD–2exchanges per day was initiated	No need for recurrent HD department visits in her frail state	Two CAPD exchanges seem to be enough to resolve her uremic symptoms
Case study 12 UK	68 years, CKD, bipolar disorder, Parkinson’s disease Transplant failed in 2019 and PD was started Loss of autonomy, but sustained PD option	CAPD increased to 3 exchanges per day, for volume control, 2 exchanges were provided by asPD team, 1 by the private carer Intensified to 4 CAPD exchanges–2 by asPD and 2 by private carers (trained by PD nurse in community)	HD was not an option for the patient, APD option was excluded due to the night wandering habit of the patient Successful dialysis was possible with asPD	AsPD responsibility can be shared between parties, paid caregivers, asPD program providers and family, in a collaborative strategy for the best interest of the patient
Case study 13 UK	Prevalent CAPD patient, with repeated hospital admissions due fluid overload in the setting of heart failure with hypotension and loss of residual kidney function	Conversion to asAPD, The patient was trained to connect and disconnect, an extra daytime exchange and cycler setup was performed by the Renal Technician	Hospital admissions ceased with regular communication by the Renal Technician and warranted advice The patient feels safe at home, now	Regular follow-up of unstable situations, whether with a community team with regular reports to the Nephrologist, as in this case, or with frequent Renal Unit visits

**Table 2:** Assisted Peritoneal Dialysis Profiles

Country	Age	Previous dialysis	assisted PD details	Nr of assisted procedures	Assistant
Austria	71	CAPD, transition to HD	APD	1	Nurses (Renal Unit)*
Finland	88	APD	CAPD	3	**Paid** Caregiver
France	85	–	CAPD followed by APD	1	Nurses (asPD program)*
Italy	85	–	Incremental CAPD	3	**Paid** Caregiver
Ireland	80	APD	APD	2	**Paid** Nursing System
Greece	78	–	CAPD	3	**Paid** Caregiver
Portugal	77	APD	APD	2	Family member
Spain	62	–	Nocturnal CAPD	2	Family member
Spain	78	HD	APD	2	Family member
Spain	19	–	APD	2	Family member
Sweden	73	–	CAPD	2	Advanced health care team*
UK	68	Transplant/CAPD	CAPD	3	**Paid** Caregiver & Community nurse*
UK	–	CAPD	APD	1	Self & Community assistance*

*Despite these examples, there is national healthcare system fund assistance in Austria, Denmark, France, Norway, Sweden and UK; and an experimental regional healthcare system fund assistance in Italy.

### Patients

Starting kidney replacement therapy for ESKD patients represents an important patient biographical breakdown that will determine their quality and span of life. In this environment and taking into consideration that ESKD is a life-threatening condition, peritoneal dialysis is in many cases the best possible treatment available and in many others the only possible treatment [5, 6].

Assisted PD has been strongly associated with older age, and although it is an important related factor, asPD is not only advocated in such condition, but actually encouraged in a broader range of non-autonomous candidates for PD. An increasing number of ESKD patients suffer from a physical or mental disability preventing adequate PD treatment implementation or performance. Frailty encompasses other dimensions that have been operationally defined by Fried et al. as meeting three out of five phenotypic criteria indicating compromised energetics: low grip strength, low energy levels, slowed walking speed, low physical activity, and/or unintentional weight loss [7].

When asPD is not available, older age and comorbid conditions can be considered contraindications for PD [8]. To respect the patient’s shared decision-making, after adequate information and presentation of available solutions, the patient might be compelled to decide for palliative treatment, as long as they do not have to spend most of the day, three days a week in a hemodialysis center often complicated by transportation issues due to disability. This is another reason to raise awareness to asPD availability, as it should be possible to offer it to these patients.

### Social environment

One of the most important and different factors between countries, and also between patient cases, is the social structure, the family structure, and relationships. Some patients have an adequate and structured social environment that, at least at the beginning, or in specific situations such as intercurrent illnesses, allows them to structure an asPD plan without any external support. Others can afford (and pay for) a professional caregiver to support the asPD therapy. Many patients, eventually, are candidates for external support for their PD treatment and the asPD should be a structured program available through the healthcare system.

### Healthcare systems

We analyzed different healthcare system models, public, private, and combined, and most of them are unable to offer adequate support to these patients. Although asPD has been available and widely used for many years in some European countries, including France, it remains unavailable or poorly utilized in many countries due to an evident lack of support from healthcare systems. In fact, AsPD is not available in many European countries because there is no funding for assistants in the community.

France has the longest and largest experience of asPD [9, 10] using private community nurses funded by the healthcare system to support patients predominantly on manual continuous ambulatory peritoneal dialysis (CAPD) 3–4 exchanges/day. Patient care is maintained by groups of 3–4 nurses with PD experience as they need to visit each patient for each exchange. They are responsible for every connection and disconnection, with the exception of APD patients who are trained to disconnect in the morning.

In many countries, though, the availability of resources for asPD patients depends more on the will and determination of healthcare professionals than on the healthcare system model. In some, even that model prevents any type of home-based assistance.

### PD therapy

Peritoneal dialysis is a self-administrated home therapy. Patients stay at home, adapting the therapy to their lifestyle, and maintaining the best possible quality of life while at the same time benefiting from the best morbidity/mortality results. These patients can lose all these benefits due to a temporary or permanent socio-sanitary situation. Peritoneal dialysis therapy is usually prescribed 7 days a week in a manual CAPD regimen, with different exchanges during the daytime or in an automatic regimen (APD) with exchanges during sleep..

Discussion made the panel of experts aware that asPD patients can benefit from a flexible and individualized prescription whereby patients, following current guidelines [11], can maintain an adequate balance between quality of life and clinical results. Incremental PD prescription allows for slower decline in residual kidney function. This feature will enable days off PD, so PD, and therefore the assistance, may only be required 5–6 days a week.

New technologies are emerging, with remote monitoring of PD treatments on APD, allowing quicker communication of treatment data with the health care team, thus improving quality of assistance, and virtual reality being used to train assistants. In Germany, some PD training teams are already using virtual reality to train PD patients. This way, the training of the trainers can be done in a resource-saving way and can be repeated as often as needed. It offers a standardized learning protocol in which individual steps can be taught separately and then put in the right order [18–20]. In Italy, the possibility to teach PD patients remotely by video training has proven to be as effective as home training, while significantly reducing the number of home visits, and can be a powerful tool as an alternative type of assistance in PD [21–23].

### Assisted peritoneal dialysis

Assisted PD is defined as PD performed at the patients’ home with the assistance of a health care technician, a community nurse, a family member, or a partner [12]. There are some well-known characteristics of asPD patients compared to patients undergoing self-care PD, such as lower risk of transfer to hemodialysis, a higher risk of death, and a lower probability of transplantation [13].

Furthermore, we have found that asPD is an adequate therapy for (1) offering PD benefits to patients previously classified as “no candidates to PD” (2) allowing kidney replacement therapy when PD is the only indicated or available therapy (3) maintaining patients on PD therapy and thus avoiding unwanted therapy discontinuation (4) providing an adequate temporary solution for ensuing patient problems.

## Discussion

The advantages of using home PD as compared to in-center HD [3] have been identified, and include preservation of residual kidney function and more cost-efficient dialysis modality, to name a few. In relatively fit older patients who can manage PD themselves, illness intrusiveness is lower on PD compared to matched patients on in-center HD [14]. Nonetheless, the inability to perform self-care is often a barrier to PD at home. Assistance is therefore needed and can be provided by a family member or paid healthcare worker; it is available in many countries worldwide and is mostly reimbursed by the healthcare system.

In 2021, this group of European nephrologists identified major barriers to the use and growth of asPD, namely overall attitudes towards PD, referring to the need for nephrology team education and/or patient education and involvement in dialysis modality decision making [3]. Secondly, the need for involvement with healthcare policymakers regarding recognition and financial support of community care is of particular importance in countries where no reimbursement for assistance exists [3]. Finally, the need for collaboration among PD units so that expertise with asPD can be shared, led to this work exposing some myths, detecting some challenges and sharing solutions found by others.

Assisted PD does not mean 4 CAPD exchanges, 7 days a week, nor does it exclude cycler, and many different prescriptions might work for our patients. For instance, supportive two-exchange asCAPD for older frail individuals can improve symptoms and can be an acceptable dialysis modality [15]. Indeed, this was adopted in case eleven (Table 1) ​​​​as a feasible alternative allowing this patient to avoid in-center HD. Tailoring the PD prescription to residual kidney function and maintaining recommended levels of small solute clearance reduces dialysis burden and is associated with higher technique survival [16].

Assisted PD does not mean assistance will be needed permanently. It can be seen as a transitory event towards individual or family caregiver autonomy, full or partial, as in the eighth case (Table 1). Initiation of home-based therapy might be made more difficult by several factors, such as the initial lack of concentration during PD training in uremic patients, the emotional fear of therapy responsibility or even the existence of a transitory physical condition. Guaranteeing initial home support could in several cases be the answer to PD choice. For example, many of our patients rely on a partner to help at first, and then feel assured that they are capable of such task. These fears are higher if the patient lives alone. Home-support can also be needed for prevalent patients, often on a temporary basis, e.g., after an acute event, partner illness, caregiver holiday [17]. Also, for some patients, assistance can be reduced to one visit a day in APD patients, in most cases only for cycler set-up, thereby reducing AsPD-related costs.

In countries without global models of asPD, family support is not the only feasible option for assistance. As in the second case (Table 1), if the patient has sufficient financial means, private care agencies can be used to provide assistance. Other options may include implementing PD training programs for the staff of nursing homes or convalescence units. Social partners may be interested in participating in smaller initiatives or for limited time periods.

Assisted PD, however, in countries without global models of asPD, will never be a reality if the nephrologist does not take it into consideration. It must be in the nephrologist’s portfolio to offer and to be talked about; then families, patients’ associations, society and government can take responsibility for assuring care. Several valuable lessons and points of learning come about through normal patients, who challenge our ability to offer them the best solution (Table 1). Table 2 clearly shows the lack of coordinated asPD programs, with family or paid private caregivers taking on the care of these patients. However, the difference in the ease of providing transport and in-center HD compared to all the efforts needed to maintain and respect the initial choice of dialysis therapy of our patients is truly amazing (Table 3).

**Table 3:** asPD take home messages

Question		Evaluate	Answer
Choice	PD	No contraindications, eliminate barriers	PD should be seen as a first dialysis option
	AsPD	Provide options	Provide initial assistance, re-evaluate capacity for performing self-care regularly
	HD less favourable	Cardiorenal and/or diabetic patients, adverse vascular access evaluation	Nephrologists’ awareness of potential complications pursuing with HD option, informed decision of the patient
Patient	Dialysis refusal	Psychologic status, and verify correct information, is PD an option?	Refusal for self-care should lead to asPD, a period on asPD might destroy myths and barriers to dialysis, and reliefs the uremic milieu
	Status	Frailty, autonomy	AsPD improves clinical status of the frail and elderly patients, but those aren´t the only ones to benefit from asPD
Prescription	Incremental asPD	Possibility of 2 home daily visits	One or two CAPD exchanges might be enough to start with
	asCAPD	Possibility of 3 home daily visits	AsPD responsibility can be shared between parties, paid caregivers, asPD program providers and family, if 3 or 4 visits are prohibitive for asPD
	asAPD	Possibility of 2 home daily visits, with eventual reduction to once/daily	The level of assistance can change, normally reducing over time
Assistant	Family member	Availability and interest	Involve the family in the decision making, although the existence of close family doesn’t mean availability for asPD,
	asPD program	Eligibility of the patient	Still the best option: an organized system of assistance–not available in all countries
	Paid caregiver	Prices and responsibility	Collaboration with Renal Units with regular communication and formation
	Community Assistant	Social partners that are able to provide for asPD	Collaboration with Renal Units with regular communication and formation
Cost	Personal	Private paid caregiver	Non-health related professionals can execute PD, as long as trained in the Renal Unit
	Country	Contrast with HD transports (health care policy)	Public care policy must change, economic studies are available, showing lower costs with asPD contrasting with in-centre HD
Availability	Ethics	Is there equity in dialysis offer?	Preserve and respect our patients’ informed option

## Data Availability

Not applicable.
